# Anti-inflammatory and antioxidant properties of *Camellia sinensis* L. extract as a potential therapeutic for atopic dermatitis through NF-κB pathway inhibition

**DOI:** 10.1038/s41598-025-86678-5

**Published:** 2025-01-18

**Authors:** Min Jung Kim, Ye Jin Yang, Ga-Yul Min, Ji Woong Heo, Jae Dong Son, Young Zoo You, Hun Hwan Kim, Gon Sup Kim, Hu-Jang Lee, Ju-Hye Yang, Kwang Il Park

**Affiliations:** 1 Department of Veterinary Physiology, College of Veterinary Medicine, Gyeongsang National University, Gazwa, Jinju, 52828 Republic of Korea; 2 Korean Medicine (KM) Application Center, Korea Institute of Oriental Medicine, 70 Cheomdan-ro, Dong-gu, Daegu, 41062 Republic of Korea

**Keywords:** Anti-inflammation, Antioxidant, Atopic dermatitis, *Camellia sinensis* L., Ultra performance liquid chromatography, Cell biology, Molecular biology, Physiology

## Abstract

Atopic dermatitis (AD) is a chronic inflammatory skin disease characterized by immune dysregulation and excessive cytokine production. This study aimed to explore the potential of *Camellia sinensis* L. water extract (CSE) as a treatment for AD by the impact of CSE on inflammatory responses in keratinocytes, particularly concerning the production of inflammatory cytokines and the modulation of signaling pathways relevant to AD pathogenesis. CSE was obtained via hot water extraction from Camellia sinensis L. Ultra-high-performance liquid chromatography (UPLC) analyzed catechin and caffeine content. Cell viability was assessed with 3-(4,5-dimethylthiazol-2-yl)-2,5-diphenyltetrazolium bromide (MTT), polyphenol and flavonoid content were determined. 2,2-diphenyl-1-picrylhydrazyl (DPPH) assay measured antioxidant activity. Enzyme-Linked Immunosorbent Assay (ELISA), western blotting, and Immunofluorescence (IF) assays examined cytokines, pathways, and protein localization, respectively. Molecular docking assessed compound binding with inflammation-related proteins. UPLC identified six CSE components including epigallocatechin (EGC) epicatechin (EC), caffeine (CF), catechin (C), epigallocatechin gallate (EGCG), and epicatechin gallate (ECG). CSE demonstrated a significant reduction in the production of inflammatory cytokines interleukin (IL)-2 and IL-6 in TNF-α/IFN-γ activated keratinocytes. Treatment with CSE inhibited the mitogen-activated protein kinase (MAPK) pathway, which resulted in decreased phosphorylation of p38, Jun N-terminal kinase (JNK), and extracellular signal-regulated kinase (ERK). Exposure of TNF-α/IFN-γ- stimulated human keratinocytes (HaCaT) cells to CSE resulted in a 200 µg/mL dependent inhibition of p65 and signal transducer and activator of transcription 1 (STAT-1) translocation from the cytosol to the nucleus, as confirmed through immunofluorescence (IF) staining. Molecular docking simulations provided insights into the underlying mechanisms of CSE action, which supported its potential as a therapeutic agent for AD. CSE might be a potential candidate for its therapeutic efficacy for inflammatory skin conditions like AD. Thus, based on this evidence, the authors suggest that CSE should be studied further for its anti-inflammatory activities and topical application in the treatment of AD.

**Supplementary Information:**

The online version contains supplementary material available at 10.1038/s41598-025-86678-5.

## Introduction

Atopic dermatitis (AD) is a chronic inflammatory skin disease^1^ characterized by severe itching, dryness, swelling, and redness. The prevalence of this disease is increasing worldwide, and despite careful epidemiological monitoring at regional and national levels, there is no consensus on the underlying causes for this rise^2^.The etiology of AD is also complex, involving an interplay among multiple immunopathological and environmental factors leading to local immune cell infiltration and symptom expression, so treatment development has proven challenging. Keratinocytes are the predominant cell type in the outer skin (epidermis) and so provide a critical barrier against biological pathogens and damaging physical stimuli. However, keratinocytes can also exacerbate inflammation by producing proinflammatory mediators in response to factors such as UV light and allergens^3,4^. These inflammatory mediators include cytokines such as interleukin (IL)-2 and IL-6, and chemokines such as thymus and activation-regulated chemokine (TARC) and regulated upon activation, normal T cell expressed and presumably secreted (RANTES)^5^. The cutaneous inflammatory response is regulated by an intricate web of genetic control mechanisms, and specific inflammatory pathways appear to be dysfunctional in AD patients including inducible nitric oxide synthase (iNOS) and cyclooxygenase-2 (COX-2)-related pathways. The expression of iNOS in upregulated in immune-activated keratinocytes, leading to enhanced production of the diffusible free radical nitric oxide (NO)^6^, whereas COX-2 is upregulated by mitogenesis, cytokines, and growth factors^7^, leading to the production of proinflammatory prostaglandins. Through these responses, iNOS and COX-2 contribute to the perpetuation and amplification of skin inflammation. Dexamethasone (Dexa), a synthetic glucocorticoid, is commonly used as a reference for anti-inflammatory studies due to its ability to suppress cytokine production and downregulate iNOS and COX-2 expression^8^. It exerts its effects by inhibiting the NF-κB and MAPKs pathways, thereby reducing inflammation and oxidative stress.

The mitogen-activated protein kinases (MAPKs) are multifunctional transducers of extracellular signals^9^ and in turn activate downstream pathways regulating cell proliferation, death, and survival^10,11^. All three major MAPKs pathways, p38, Jun N-terminal kinase (JNK), and extracellular signal-regulated kinase (ERK), were phosphorylated (activated) in human keratinocytes (HaCaT) cells upon stimulation with the proinflammatory factors tumor necrosis factor (TNF)-α and interferon (IFN)-γ^11,12^, Blocking MAPK pathways reduced inflammatory cytokine production^12^. These MAPKs pathways are downregulated by negative feedback loops under conditions of excessive activity, thereby adjusting the proinflammatory/immunoregulatory balance and preventing chronic inflammation. In addition to MAPKs, the signaling factors NF-kappa B (NF-κB) and signal transducer and activator of transcription 1 (STAT-1) are activated (phosphorylated) under proinflammatory conditions. Therefore, controlling MAPKs, NF-κB, and STAT-1 activities is essential for preventing pathological skin inflammation^13^. Current AD therapies include corticosteroids to suppress inflammation and antihistamines to suppress allergic reactions, but the long-term use of these agents can have adverse effects^14^, necessitating the development of safer alternatives. Natural extracts of plants are among the most promising alternatives as they possess numerous relevant bioactivities and demonstrate good safety profiles^15^.

The young foliage of the flowering shrub *Camellia sinensis* L. has long been used to produce white tea, yellow tea, green tea, oolong, and dark tea with diverse health benefits, including suppression of inflammation^16^, enhancement of microvascular elasticity^16^, antioxidant capacity^17^, photo resistance, anticellulite and slimming effects, and improvements in skin and hair condition^16^. Despite these numerous benefits, little research has been conducted on the potential therapeutic efficacy of *Camellia sinensis* L. water extract (CSE) for AD.

This study aims to evaluate the anti-inflammatory and antioxidant properties of CSE in TNF-α/IFN-γ-activated HaCaT cells, a well-studied in vitro model of AD, and to further examine the contributions of MAPKs, NF-κB, and STAT-1 signaling pathways. Therefore, this study investigated the effect of CSE on AD and confirmed its potential as a candidate compound for the AD treatment based on signaling pathways.

## Results

### Evaluation of CSE cytotoxicity in HaCaT cells

**Fig. 1:**
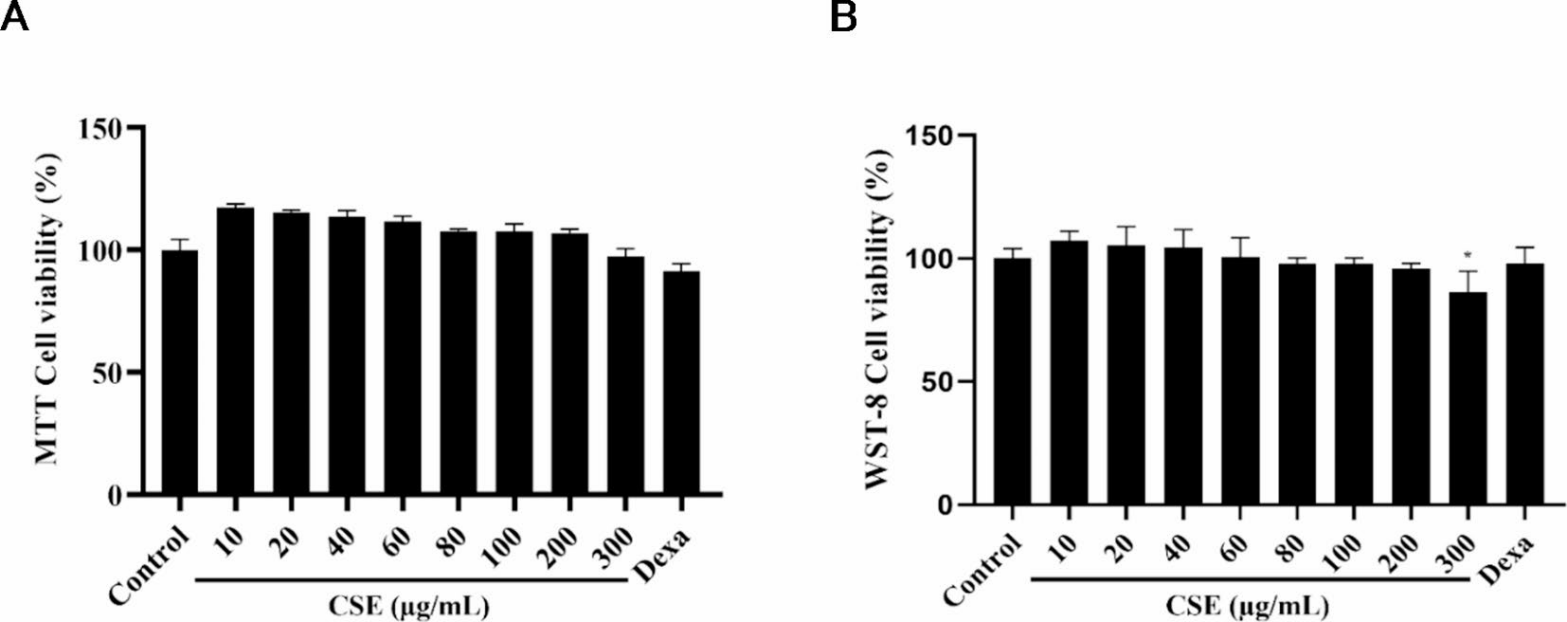
Effect of CSE on HaCaT keratinocyte viability. (**A**) MTT assay and (**B**) WST-8 assay results showing the effect of CSE on HaCaT cell viability. HaCaT cells were treated with varying concentrations of CSE (0–300 µg/mL) for 24 h. CSE did not substantially reduce HaCaT keratinocytes by 200 µg/mL. Dexa:10 µg/mL. The results obtained from three independent experiments were expressed as means ± standard deviation (SD), compared with the control group. *p < 0.05 vs. control group.

The cytotoxicity of CSE was then evaluated using the 3-(4,5-Dimethylthiazol-2-yl)-2,5-Diphenyltetrazolium Bromide (MTT) and WST-8 viable cell counting assay. Incubation of HaCaT cells in CSE at concentrations of 10, 20, 40, 60, 80, 100, and 200 µg/mL for 24 h had no substantial effects on viable cell number (Fig. 1), while reduced viability was observed at 300 µg/mL (*p* < 0.05). Thus, concentrations of 50, 100, and 200 µg/mL were used in subsequent experiments examining effects on inflammatory responses and underlying signaling pathways.

### Effects of CSE on inflammatory mediators in TNF-α/IFN-γ-stimulated HaCaT cells

**Fig. 2:**
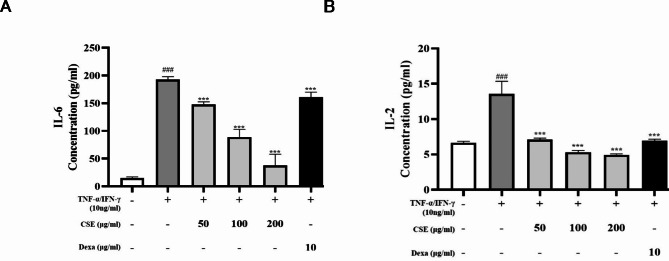
Dose-dependent suppression of TNF-α/IFN-γ-stimulated proinflammatory release by CSE. HaCaT cells were pretreated with TNF-α/IFN-γ (each 10 ng/mL) for 1 h and then stimulated with CSE for 24 h. Release concentration of (**A**) IL-6 and (**B**) IL-2 were measured by ELISA. Results are presented as mean ± standard error of the mean (SEM) of three independent experiments. ###*p* < 0.001 vs. TNF-α/IFN-γ-stimulated group; ***p < 0.001 vs. control group. Dexa: dexamethasone (positive control).

The effects of CSE on inflammatory mediator levels were evaluated by using enzyme-linked immunosorbent assay (ELISA). The production of IL-6 and IL-2 were significantly higher in the TNF-α/IFN-γ-stimulated control group compared to the normal group (*p* < 0.001). However, these levels suppressed following CSE treatment in a dose-dependent manner (*p* < 0.001), suggesting that CSE effectively reduces inflammation in this cellular model. Dexa significantly reduced the expression of IL-6 and IL-2 compared to the TNF-α/IFN-γ induction group (*p* < 0.001) Dexa was less effective than the CSE group in reducing the expression of IL-6 and IL-2 (Fig. 2).

### Effects of CSE on protein expression levels of iNOS and COX-2 in HaCaT cells stimulated with TNF-α/IFN-γ

**Fig. 3:**
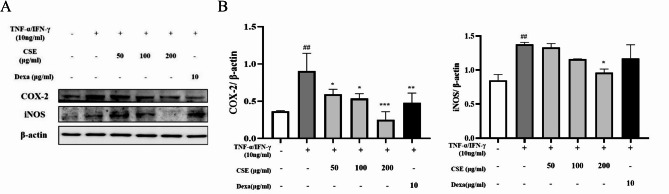
Inhibition of TNF-α/IFN-γ-stimulated iNOS and COX-2 expression in HaCaT cells by CSE. HaCaT cells were incubated with after a 1h pretreatment with TNF-α/IFN-γ (10 ng/mL each) and then stimulated with CSE for 24 h. (**A**) A typical Western blot showing COX-2 and iNOS expression under the indicated treatment conditions. The expression β-actin was measured as the gel loading control. (**B**) Bar graphs show the densities of the COX-2 band and iNOS band normalized to β-actin band density. Results are presented as the mean ± SD of three independent experiments. *p < 0.05, **p < 0.01, ***p < 0.001 vs. TNF-α/IFN-γ-stimulated group; ##*p* < 0.01 vs. control group. Dexa: dexamethasone (positive control).

The expression levels of iNOS and COX-2, the enzymes producing NO and prostaglandin E_2_ (PGE_2_), respectively, were markedly elevated in HaCaT cells stimulated with TNF-α/IFN-γ for 24 h as determined by western blotting, while cotreatment with CSE dose-dependently suppressed both responses (Fig. 3). However, CSE was substantially more effective at suppressing the rise in COX-2 expression *(p* < 0.001) than iNOS expression (*p* < 0.05). Dexa significantly reduced the expression of COX-2 compared to the TNF-α/IFN-γ induction group (*p* < 0.01). However, while the expression of iNOS decreased relative to the TNF-α/IFN-γ induction group, the difference was not statistically significant (Fig. 3).

### Treatment with CSE dose-dependently suppressed TNF-α/IFN-γ-stimulated STAT-1 and NF-κB (p65) phosphorylation in HaCaT cells

**Fig. 4:**
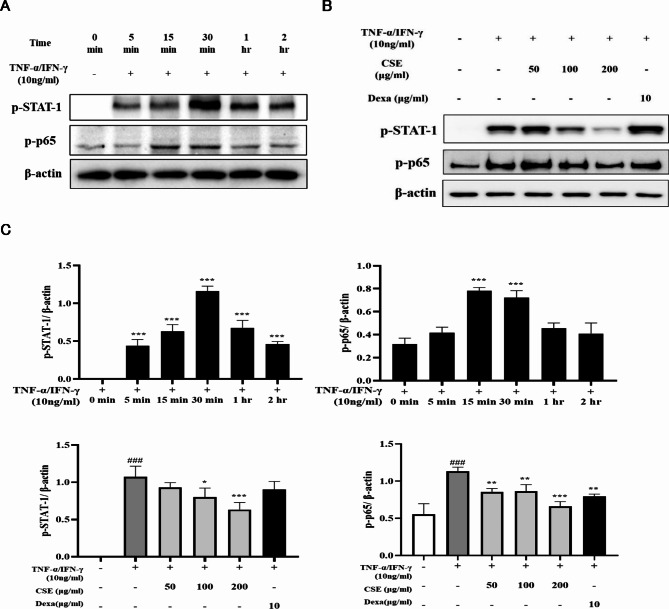
Inhibition of TNF-α/IFN-γ-stimulated NF-κB and STAT-1 signaling in HaCaT cells by CSE. (**A**) Typical Western blots showing the time courses of NF-κB and STAT-1 phosphorylation induced by TNF-α/IFN-γ. (**B**) Treatment with TNF-α/IFN-γ induced a transient rise in p-p65, and p-STAT-1 expression levels peaked at around 30 min. These responses were suppressed dose-dependently by 1 h of CSE pretreatment. (**C**) Bar graphs of p-p65 and p-STAT-1 band densities relative to β-actin band density (the gel loading control). Treatment with TNF-α/IFN-γ for 30 min induced transient increases in p-p65 and p-STAT-1 expression levels that peaked. These increases in phosphorylation were dose-dependently reversed by CSE pretreatment. All values are presented as the mean ± SD of three independent experiments. *p < 0.05, **p < 0.01, ***p < 0.001 vs. TNF-α/IFN-γ-stimulated group; ###*p* < 0.001 vs. control group. Dexa: dexamethasone.

Western blotting experiments were then conducted to assess the potential contributions of STAT-1 and NF-κB signaling to TNF-α/IFN-γ-evoked inflammatory responses in HaCaT cells^19^ and suppression by CSE. Like MAPKs responses, STAT-1 phosphorylation levels rose transiently during TNF-α/IFN-γ treatment, peaking at 30 min *(p* < 0.001) (Fig. 4A). Further, these responses were dose-dependently suppressed by CSE. Dexa significantly reduced the expression of p-p65 compared to the TNF-α/IFN-γ induction group (*p* < 0.01). However, while the expression of p-STAT1 decreased relative to the TNF-α/IFN-γ induction group, the difference was not statistically significant (Fig. 4B).

### Effects of CSE on the phosphorylation of MAPKs in HaCaT cells stimulated with TNF-α/IFN-γ

**Fig. 5:**
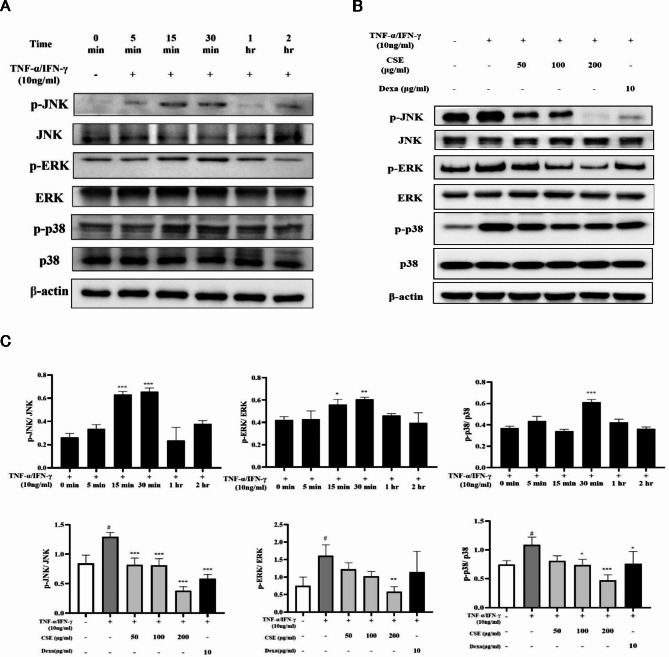
Inhibition of MAPKs phosphorylation/activation by CSE in TNF-α/IFN-γ-stimulated HaCaT cells. (**A**) Typical Western blots showing the time course of MAPKs phosphorylation (p-JNK, p-ERK, and p-p38) in HaCaT cells during stimulation with TNF-α/IFN-γ (10 ng/mL each) alone and following pretreatment with CSE. (**B**) Treatment with TNF-α/IFN-γ induced a transient rise in p-JNK, p-ERK, and p-p38 expression levels that peaked at around 30 min. These responses were suppressed dose-dependently by 1 h of CSE pretreatment. (**C**) Bar graphs of p-JNK, p-ERK and p-p38 band densities relative to total form band density (the gel loading control). All values are the mean ± SD of three independent experiments. *p < 0.05, **p < 0.01, ***p < 0.001 vs. TNF-α/IFN-γ-stimulated group; #*p* < 0.05 vs. control group. Dexa: dexamethasone (positive control).

To examine the potential contributions of MAPK signaling pathways in the inflammatory response and their suppression by CSE, HaCaT cells were exposed to TNF-α/IFN-γ (10 ng/mL each)^18^ in the presence and absence of CSE, and MAPKs phosphorylation activation levels estimated periodically by Western blotting (Fig. 5A). Significant elevations of ERK, JNK, and p38 phosphorylation were detected after 30 min in TNF-α/IFN-γ-treated HaCaT cells, and all these responses were partially reversed by CSE (*p* < 0.001) (Fig. 5B). Dexa significantly reduced the expression of p-JNK and p-p38 compared to the TNF-α/IFN-γ induction group (*p* < 0.001 and *p* < 0.01). However, while the expression of p-ERK decreased relative to the TNF-α/IFN-γ induction group, the difference was not statistically significant (Fig. 5C). Dexa was less effective than the CSE 200 µg/mL group in reducing the expression of phosphorylated MAPKs (p-JNK, p-ERK, and p-p38) (Fig. 5B,C), suggesting that CSE at higher concentrations may have a stronger inhibitory effect on MAPK activation in this experimental.

### Treatment with CSE dose-dependently suppressed TNF-α/IFN-γ-induced nuclear translocation of NF-κB and STAT-1 in HaCaT cells

**Fig. 6:**
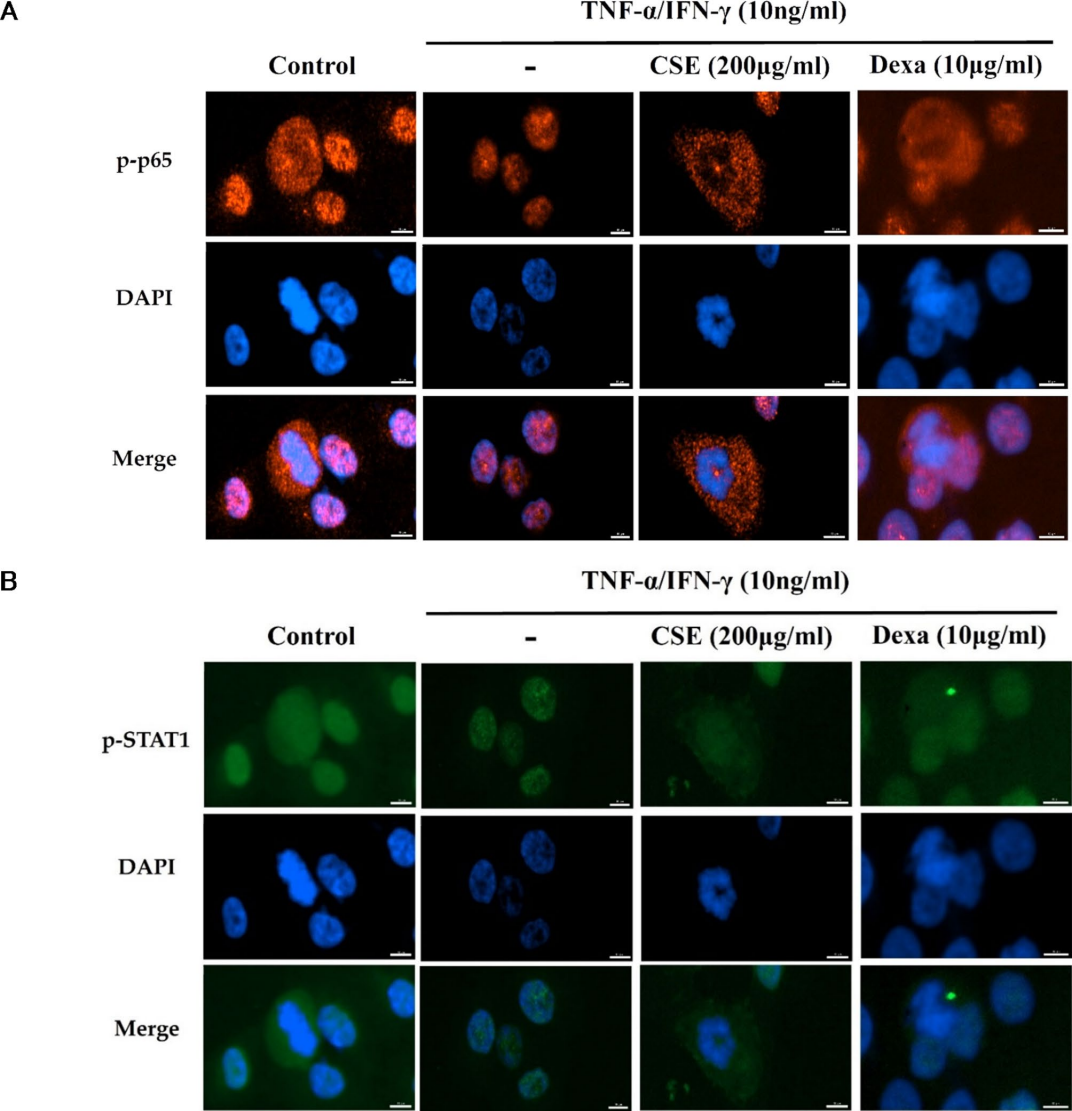
Inhibition of TNF-α/IFN-γ-stimulated p-p65 and p-STAT-1 nuclear translocation by CSE. (**A**) Immunofluorescence images of HaCaT keratinocyte cells stained with an antibody against p-p65 (red) and counterstained with DAPI (blue). (**B**) Immunofluorescence images of HaCaT cells stained with an antibody against p-STAT-1 (green) and counterstained with DAPI (blue). Treatment with TNF-α/IFN-γ for 30 min enhanced nuclear translocation as evidenced by increased dual staining with DAPI in merged images. Translocation of both factors was blocked by 1 h preincubation with 200 µg/mL CSE. After the indicated treatments, cells were fixed in 4% formaldehyde, stained with the indicated primary antibody, and counterstained with DAPI. Merged images indicate colocalization. scale bar = 10 μm. Dexa: dexamethasone (positive control).

The transcriptional activities of NF-κB and STAT-1 require both phosphorylation and translocation into the nucleus, so we further examined if these events were also influenced by CSE using immunofluorescence imaging and nuclear counterstaining. As expected, the active p-p65 and p-STAT-1 were translocated into the nucleus 4’,6-diamidino-2-phenylindole ((DAPI))-stained region) during TNF-α/IFN-γ treatment, and this translocation was dose-dependently inhibited by CSE (Fig. 6).

### Quantification of total phenolic and flavonoid contents in CSE

Total polyphenol and total flavonoid contents of CSE based on colorimetric assays are presented in Table 1. The total polyphenol content was 154.9 ± 2.95 GAE mg/g and the total flavonoid content was 85.49 ± 5.31 QC mg/g.

**Table 1:** Total phenolic and flavonoid contents of CSE.

Items	Concentration
Total polyphenol (GAE mg/g)^1^	154.9 ± 2.95
Total flavonoid (QE mg/g)^2^	85.49 ± 5.31

^1^Total polyphenol content is expressed as gallic acid equivalents (GAE).

^2^Total flavonoid content is expressed as quercetin equivalent (QE).

### Total antioxidant capacity of CSE

**Fig. 7:**
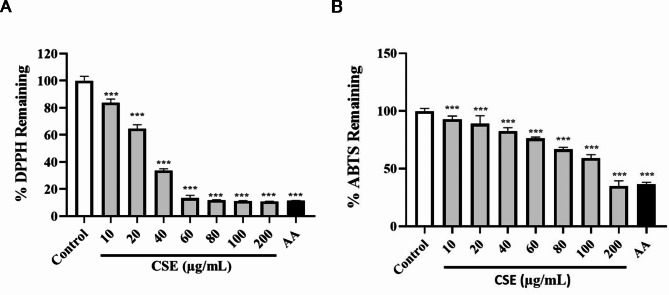
Concentration-dependent DPPH and ABTS free radical remaining by CSE. Results are the mean ± standard deviation (SD) of three independent experiments. *** *p* < 0.001 vs. the CSE control group. Ascorbic acid (AA): 100 µg/mL ascorbic acid (positive control).

The total antioxidant capacity of the CSE sample used for experiments (measured as the amount of free radical DPPH remaining after incubation) was 13.26 ± 1.82% at 60 µg/mL (*p* < 0.001), 11.85 ± 0.05% at 80 µg/mL (*p* < 0.001), 11.09 ± 0.06% at 100 µg/mL (*p* < 0.001), and 10.84 ± 0.11% at 200 µg/mL (*p* < 0.001). At a concentration of 60 µg/mL or higher, the total antioxidant capacity was roughly equivalent to that of the positive control Ascorbic acid (AA) (11.8% at 100 µg/mL) (*p* < 0.001) (Fig. 7A). The total antioxidant capacity of the CSE sample used for experiments (measured as the amount of free radical ABTS that remained after incubation) was 34.98 ± 4.10% at 200 µg/mL (*p* < 0.001), The total antioxidant capacity was roughly equivalent to that of the positive control AA (36.7% at 100 µg/mL) (*p* < 0.001) at a concentration of 200 µg/mL or higher (Fig. 7B).

### Ultra performance liquid chromatography (UPLC) analysis of CSE components

**Fig. 8:**
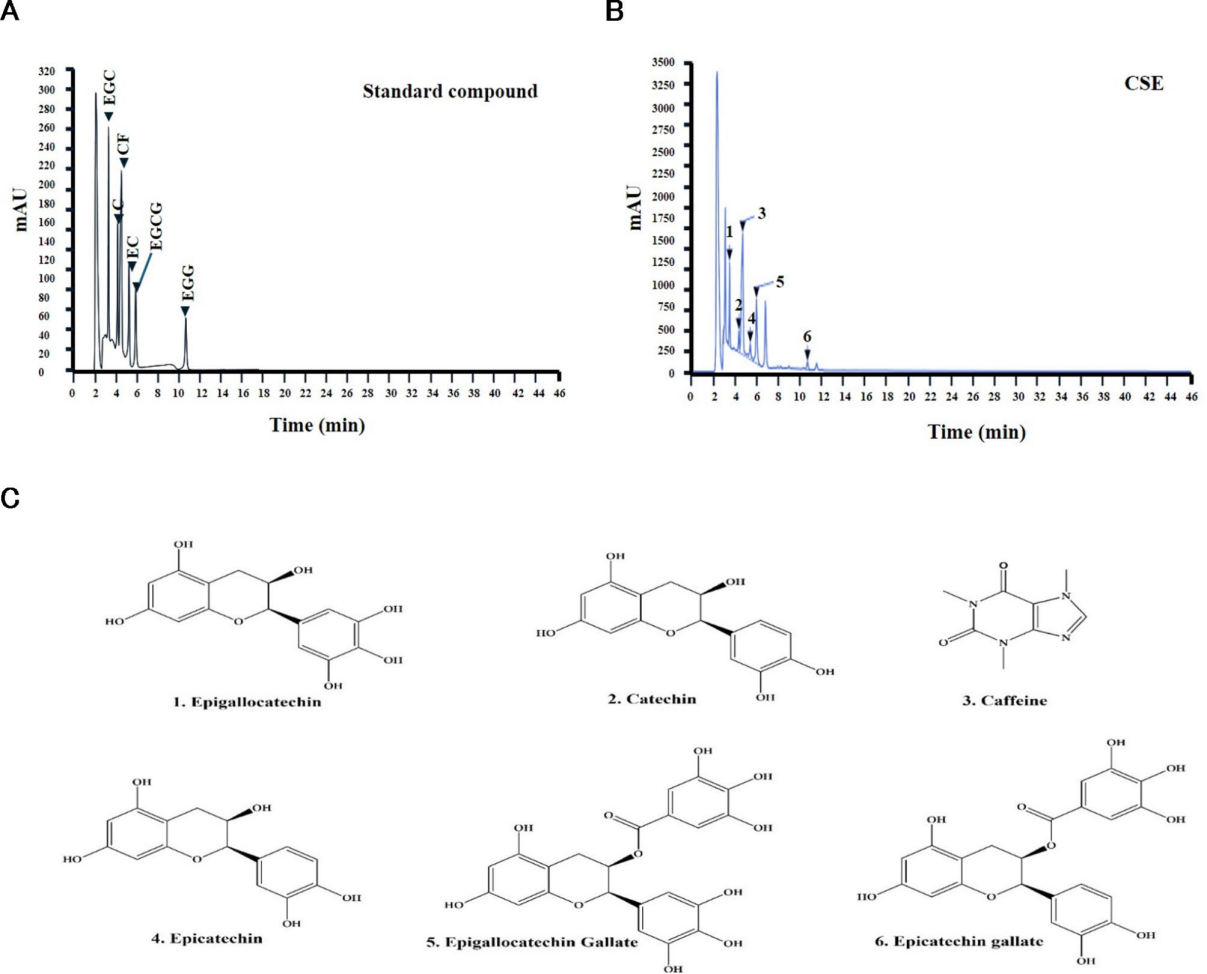
Chromatograms identifying five catechins and caffeine in CSE powder based on retention time matching with known standards. Eluents were monitored at 240 nm. Shown are typical UPLC chromatograms of (**A**) standard compounds and (**B**) a CSE sample. (**C**) Molecular structures of the identified compounds.

To identify the bioactive ingredients of CSE, 1 mg of powder was mixed with 1 mL of 70% methanol, and the extract was analyzed using high-performance liquid chromatography. Five distinct catechin compounds, epigallocatechin (EGC), catechin (C), epicatechin (EC), epigallocatechin gallate (EGCG), and epicatechin gallate (ECG) as well as caffeine (CF), were successfully identified (Peaks 1–6, Fig. 8A,B). The retention times of these compounds were as follows: 3.46 min (EGC), 4.33 min (C), 4.69 min (CF), 5.41 min (EC), 6.036 min (EGCG), and 10.806 min (ECG). Five distinct catechin compounds EGC (C15H14O7), C (C15H14O6), EC (C15H14O6), EGCG (C22H18O11), and ECG (C22H18O10) as well as CF (C8H10N4O2), were successfully identified. Their molecular structures are depicted in Fig. 8C.

**Table 2:** UPLC data of the catechin and caffeine compounds in CSE.

Peak no.	Retention time (min)	Compounds	Content (%)
1	3.46	Epigallocatechin	2.170 ± 0.036
2	4.33	Catechin	0.882 ± 0.040
3	4.69	Caffeine	3.615 ± 0.147
4	5.41	Epicatechin	0.983 ± 0.299
5	5.98	Epigallocatechin gallate	4.179 ± 0.235
6	10.79	Epicatechin gallate	0.715 ± 0.012
Total contents (%)		8.929 ± 0.622	

Notably, all six compounds were identified at high chromatographic resolution with strong reproducibility. Comparisons with standard curves indicated that the CSE contained 3.615% CF and 5.764% total catechin by weight, with EGCG accounting for the largest fraction. The contents of all catechins and caffeine are listed in Table 2.

### Molecular docking simulations of catechin and caffeine with NF-кB

**Fig. 9:**
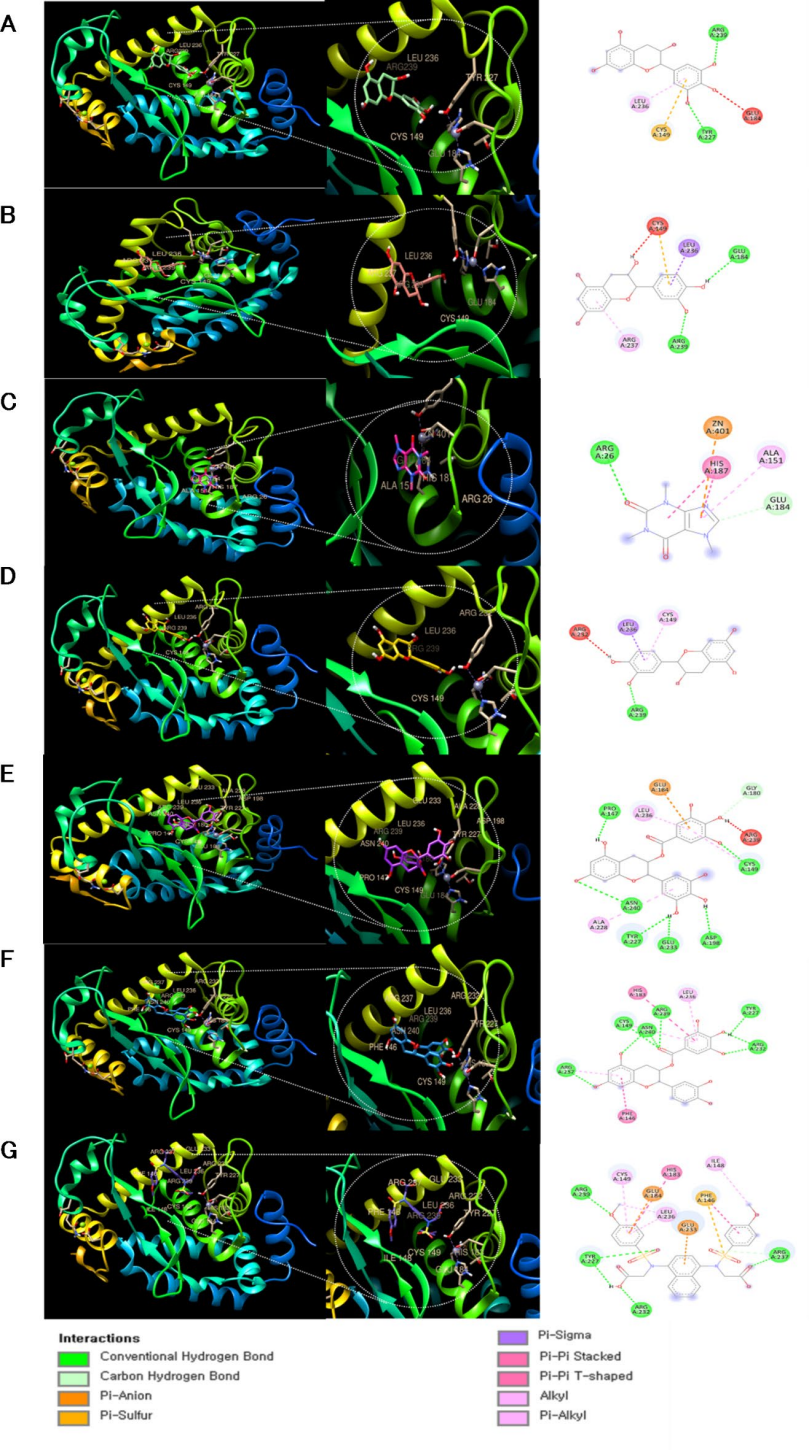
Molecular docking analysis of NF-кB and polyphenolic compounds in CSE. The 3D structure of NF-кB bound efficiently to (**A**) EGC, (**B**) C, (**C**) CF, (**D**) EC, (**E**) EGCG, and (**F**) ECG, as well as to the known inhibitor (**G**) CPUY192018 (positive control).

To examine these anti-inflammatory effects of CSE at the molecular level, we conducted ligand-protein docking simulations using UCSF Chimera (Fig. 9). All six catechin compounds examined occupied the active site of NF-кB. A comparison of binding energy scores Table 3 suggested that the two polyphenolic compounds ECG and EGCG bind with the highest affinity. Binding energies were higher than that of the known NF-кB inhibitor CPUY2018.

**Table 3:** Binding energies of selected CSE components with the NF-кB complex according to docking simulations.

Binding ligand	Amino acid residue that interacts	Docking score
Epigallocatechin	ARG239, LEU236, CYS149, TYR227, GLU184	−7.4 kcal/mol
Catechin	CYS149, LEU236, ARG239, ARG237, GLU184	−7.2 kcal/mol
Caffeine	ARG26, ZN401, HIS187, ALA151, GLU184	−5.5 kcal/mol
Epicatechin	ARG252, LEU236, CYS149, ARG239	−7.0 kcal/mol
Epigallocatechin gallate	GLU184, LEU236, GLY180, ARG239, CYS149, ASN240, ALA228, TYR227, GLU233, ASP198	−7.9 kcal/mol
Epicatechin gallate	HIS 183, ARH239, LEU236, TYR227, ARG232, CYS149, ASN240, ARG237, PHE146	−8.4 kcal/mol
CPUY192018	ARG239, CYS149, GLU184, HIS183, LEU236, TYR227, ARG232, GLU233, PHE146, ILE148, ARG237	−7.8 kcal/mol

## Discussion

AD can substantially reduce quality of life and is increasing in prevalence across the globe. Current treatments such as steroids are effective but carry long-term health risks. The etiology of AD is multifactorial^20^ and numerous alternative approaches have been investigated for managing skin inflammation and restoring skin barrier function^21^.

Keratinocytes are the predominant cell type in the outer skin, and in addition to forming a physical barrier to pathogens, these cells function as sources of danger signals that can trigger inflammatory responses. AD is caused by excessive immune cell recruitment to skin lesion sites, leading to activation of cells and the local release of inflammatory cytokines that sustain the inflammatory response^22^.

Administration of CSE to cultured cells reduced expression and release of the proinflammatory cytokines IL-2 and IL-6 in response stimulation by TNF-α and IFN-γ, in accord with previous studies^22,23^ (Fig. 2). Further, these anti-inflammatory effects were observed at concentrations well below the cytotoxicity threshold (about 300 µg/mL), highlighting the safety of this anti-inflammatory strategy (Fig. 1).

AD is strongly associated with activation of NF-κB, STAT-1, and MAPKs signaling pathways in HaCaT cells^24,25^. Stimulation by TNF-α phosphorylates the NF-κB subunit p65 and degrades the regulatory subunit inhibitor of nuclear factor kappa B (IκBα), resulting in translocation of p-p65 to the nucleus^26^ and activation of genes encoding proinflammatory chemokines and cytokines such as IL-2 and IL-6. In the current study, CSE inhibited the expression of these downstream cytokines and suppressed both the phosphorylation and nuclear translocation of p65 in response to TNF-α/IFN-γ stimulation (Figs. 5, 4 and 6). Therefore, inhibition of NF-κB transcriptional activity is likely crucial to the anti-inflammatory efficacy of CSE.

Plant extracts such as CSE contain a variety of compounds with distinct and known bioactivities, including polyphenols, flavonoids, alkaloids, and amino acids^27^. Total polyphenols and flavonoids assays revealed substantial total levels of polyphenols (154.9 µg/mL) and flavonoids (82.85 µg/mL) (Table 1). Polyphenols and flavonoids might account for the high antioxidant capacity of CSE as these compounds can directly scavenge free radicals and chelate metal ions. Additionally, the ABTS and DPPH assays showed a decrease in radical levels dependent on the concentration, indicating that CSE has the ability to reduce oxidative stress. A concentration of 200 µg/mL of CSE exhibited free radical scavenging capabilities comparable to or higher than 100 µg/mL of the naturally occurring antioxidant AA (Fig. 7). The capacity of CSE to reduce free radicals indicates a protective function in combating oxidative stress-induced harm, which is essential in the prevention of skin conditions like atopic dermatitis and premature aging.

In addition to direct free radical scavenging and metal chelation, polyphenols and flavonoids are known to act as endogenous antioxidant inducers. Collectively, the enhanced cellular antioxidant capacity conferred by CSE treatment might help prevent pathological skin inflammation.

Furthermore, chromatographic analysis confirmed the presence of five catechins (polyphenols) as well as caffeine (an alkaloid) in CSE (Fig. 8; Table 2). In fact, we further demonstrated that multiple catechin components bind to NF-κB, with ECG and EGCG showing particularly strong affinity (Fig. 9; Table 3). Through the molecular docking of the representative inflammation-related receptors NF-кB and compounds, it was confirmed that the components of CSE have significantly higher binding scores in terms of structural combination polyphenolic. The presence of polyphenolic compounds in CSE indicates that CSE could be a valuable therapeutic agent for treatment of skin inflammation due to of its antioxidant and anti-inflammatory properties. The molecular structure of these polyphenolic compounds enhances antioxidant efficacy in various inflammation-related pathways by providing structural affinity.

This study examined the impact of CSE on skin inflammation and anti-atopic dermatitis utilizing the HaCaT keratinocyte cell line. HaCaT cells are commonly utilized in the investigation of skin biology. However, they fail to completely mirror the complexity of authentic physiological skin conditions. Consequently, in order to ascertain the potential therapeutic effects of CSE for Alzheimer’s disease, it is essential to validate the findings using in vivo animal models or human clinical trials. Despite these limitations, CSE shows various anti-inflammatory effects that can effectively alleviate the symptoms of AD, which confirming its potential as a candidate for AD treatment.

## Methods

### Preparation of CSE

The CSE, used in this study was prepared by incubating *Camellia sinensis* L. leaves in 10 times the weight of warm water (60 °C) for 3 h in an extractor (WHM12074, Large capacity steel case heating mantle, Daihan science). Our plant study complies with relevant institutional, national and international guidelines and legislation. The leaves of *Camellia sinensis* L. were procured from the Yeong Cheon Oriental Herbal Market, located in Yeong Cheon, Korea. The specimens utilized for verification were young leaves of Camellia *sinensis* L. and were identified by Professor Young Woo Kim from the School of Korean Medicine at Dongguk University, Gyeongju, Korea. A specimen designated as voucher 202,206 was deposited in the herbarium of the appropriate Korea Institute of Oriental Medicine. This process was repeated 3 times, and the raw extract was then pooled, concentrated using a vacuum concentrator (EYELA, Tokyo, Japan, yield 2.1%), and stored at − 20 °C. For individual experiments, CSE powder (10 mg) was dissolved in 1 mL of distilled water and cleared by passage through a 0.22 µm pore filter.

### Cell culture

The HaCaT cells, a human keratinocyte cell line, used in the investigation were provided by the Korea Institute of Oriental Medicine and purchased from Cell Lines Service (Eppelheim, Deutschland, Germany). The cell culture media, Dulbecco’s Modified Eagle’s Medium supplemented with 10% heat-inactivated fetal bovine serum (Thermo Fisher Scientific, MA, USA) and 1% penicillin/streptomycin was purchased from Gibco (Thermo Fisher Scientific, MA, USA). For the investigation, cells were used after being passaged at least three times to ensure stability and minimize potential variability from cells at lower passage numbers.

### MTT cell viability assay

Cell viability was evaluated using MTT (#298-93-1, Duchefa Biochemie, Haarlem, Netherlands) assay. HaCaT cells (2 × 10^4^ cells/well) were seeded in 96-well plates and stabilized for 24 h. Cells were treated with CSE at various concentrations, and cell viability was subsequently evaluated 3 times. The HaCaT cells were incubated with concentration from 10 µg/mL to 300 µg/mL of CSE for 24 h. The wells were gently washed, and the medium replaced with MTT reagent for 2 h. The absorbance was then measured with a Mobi-Microplate Spectrophotometer (MicroDigital, Korea) at 565 nm.

### WST-8 cell viability assay

HaCaT cells were seeded at 2 × 10^4^/well in 96-well plates, incubated for 24 h, and then treated with from 10 µg/mL to 300 µg/mL of CSE for 24 h. Quanti-MAX WST-8 Cell viability Assay Kit reagent (#QM5000, Biomax, Korea) was added to 10µL media and incubated at 37 °C for 1 h. The absorbance of the WST-8 solution was read at 450 nm and each sample was analyzed using a Mobi-Microplate Spectrophotometer (MicroDigital, Korea).

### ELISA

The IL-2 (#A35603, Invitrogen, Waltham, MA, USA) and IL-6 (#A35573, invitrogen, Waltham, MA, USA) secreted from the cells were measured using respective ELISA kits. Cytokine was measured according to respective manufacturer’s instructions. After treating CSE and inflammatory cytokines for 24 h, the medium was transferred to a 1 ml tube and centrifuged at 3000 rpm and 4 °C for 15 min. The supernatant was transferred to a new 1 mL tube in preparation for measuring IL-2 and IL-6 levels. All reagents were equilibrated to room temperature before use. The wash buffer was prepared by diluting the concentrated wash solution according to the manufacturer’s instructions. The IL-2 and IL-6 standards were prepared by dissolving them as instructed and creating a series of dilutions using the provided standard diluent. Each supernatant was dispensed along with 50 µL of standards into the wells of a pre-coated 96-well microplate. An equal volume (50 µL) of biotinylated anti-IL-2 and IL-6 detection antibody was added to each well to facilitate specific antigen–antibody binding. The plate was incubated at room temperature for 1 h and washed five times with wash buffer to remove unbound components. Subsequently, 100 µL of streptavidin-HRP solution was added to each well and incubated for 30 min at room temperature. After washing, 100 µL of TMB substrate solution was added to each well and incubated for 20–30 min in the dark. The reaction was terminated by adding 50 µL of stop solution, and absorbance was immediately measured at 490 nm using a microplate spectrophotometer (Mobi, Korea). Cytokine concentrations (in pg/mL) were extrapolated from IL-2 and IL-6 standard curves constructed over the range from 0 to 2000 pg/mL.

### Western blotting

Cell lysate proteins (20 µg samples) were separated by 10% sodium dodecyl sulfate polyacrylamide gel electrophoresis and transferred onto polyvinylidene difluoride membranes (Millipore, Billerica, MA, USA). Membranes were blocked by incubation in 5% skim milk for 30 min at room temperature. The membranes utilized in the Western blotting analysis were trimmed to correspond with the molecular weight of the protein prior to being subjected to hybridization with the antibody. Then the membranes were incubated with primary antibodies against COX-2 (#12282), iNOS (#13120), p-ERK (#9101), p-p38 (#8690), p-JNK (#4668), p-p65 (#3031), p-STAT-1(#9167) (1:1000, Cell Signaling Technology, Danvers, MA, USA), and β-actin (#ATGA0570 1:1000, NKMAX, Korea) overnight at 4 °C and were washed thrice with Tris-buffered saline plus Triton-X (TBST) (10 min per wash) and then incubated in secondary antibody goat anti-mouse IgG-HRP Conjugate (#1706516; 1:5000, BIO-Rad) and goat anti-rabit IgG-HRP Conjugate (#1706515; 1:5000, BIO-Rad) for 2 h at room temperature. Immunoreactivity was detected using SuperSignal™ West Pico PLUS Chemiluminescent Substrate (Cat. no 46640; Thermo Fisher Scientific, Waltham, MA, USA). Expression of β-actin was measured as the gel loading control. The intensities of target and control protein bands were measured using ImageJ (version. 18.0).

### Immunofluorescence assay

Cells were seeded at 5 × 10^4^ cells/well in 4-well plates (SPL, Korea), incubated with CSE or dexamethasone for 1 h, and then stimulated with TNF-α/IFN-γ (10 ng/mL each) for 30 min in the continued presence of CSE or dexamethasone. Cells were fixed with 4% paraformaldehyde in PBS, blocked with 3% goat serum (Gibco, Grand Island, NY, USA) for 1 h, and incubated with 200 µL/well anti-p65 rabbit polyclonal antibody (1:100, Invitrogen, Waltham, MA, USA) and anti-STAT-1 mouse polyclonal antibody (1:100, Invitrogen) at 4°C overnight. Fixed cells were then incubated with Alexa Fluor 488-conjugated goat anti-rabbit secondary antibody (1:100) and Alexa Fluor 568-labeled goat anti-mouse secondary antibody (1:100) (both from Life Technologies, Carlsbad, CA, USA) for 2 h at room temperature. The wells were washed with PBS and the remaining fluid carefully removed. Cultures were counterstained with one drop of mounting solution containing DAPI, sealed under cover glass (DWK Life Sciences, China), and examined using an automated live cell imager (LIONHEART™ FX, BioTek, Winooski, Vermont, USA) with dedicated analysis software (Gen5 ™ 3.0, BioTek, Vermont, USA).

### Total polyphenol content assessment

The total phenolic content of CSE was determined using the method described by Singleton and Rossi with some modifications^28^. 1 mg/mL gallic acid solution was prepared using distilled water and serially diluted to generate a calibration curve. For each 100 µL of the diluted gallic acid solutions, 500 µL of 2 N Folin’s phenol reagent and 400 µL of 7.5% Na₂CO₃ were added and mixed thoroughly. Similarly, 100 µL of a 2 mg/mL CSE was mixed with 500 µL of 2 N Folin’s phenol reagent and 400 µL of 7.5% Na₂CO₃. The mixtures were then dispensed into a 96-well plate and incubated in the dark at room temperature for 1 h. Absorbance was measured at 750 nm using a microplate reader (BioTek Synergy H1). The total phenol content is expressed as milligrams of gallic acid (gallic acid equivalents (GAE), 0–300 µg/mL) per gram of extract (mg GAE/g extract).

### Total flavonoid content assessment

Total flavonoid content was measured using the aluminum chloride-based colorimetric method described by Aiyegoro and Okoh^29^. 1 mg/mL quercetin solution was prepared using 100% methanol and serially diluted to generate a calibration curve. For each 100 µL of the diluted quercetin solutions, 860 µL of 80% ethanol, 20 µL of 10% AlCl₃, and 20 µL of 1 M potassium acetate were added and mixed thoroughly. Similarly, 100 µL of a 2 mg/mL CSE was mixed with 860 µL of 80% ethanol, 20 µL of 10% AlCl₃, and 20 µL of 1 M potassium acetate. The mixtures were then dispensed into a 96-well plate and incubated at room temperature for 40 min. Absorbance was measured at 415 nm using a microplate reader (BioTek Synergy H1). The total flavonoid content was calculated based on the calibration curve. Quercetin (quercetin equivalents (QE), 0–300 µg/mL) served as a standard, and the results are expressed as mg QE/g extract.

### 2,2-Diphenyl-1-picrylhydrazyl (DPPH) assay

The antioxidant capacity of the CSE was evaluated using the DPPH (2,2-diphenyl-1-picrylhydrazyl) assay, which measures the ability of antioxidants to react with the stable free radical DPPH, leading to a change in its spectral properties. The DPPH reagent (Thermo Fisher Scientific, MA, USA) was diluted with 100% methanol to adjust its concentration to achieve an absorbance of 1.00 ± 0.02 at 517 nm. More specifically a 0.0913 mM DPPH stock solution was prepared by dissolving 0.0036 g of DPPH in 100 mL of methanol. Each day, fresh working solutions were prepared by diluting the stock solution with methanol to maintain an absorbance of 1.00 ± 0.02 at 517 nm. CSE was prepared by diluting a 100 mg/mL stock solution with 100% methanol to obtain final concentrations ranging from 0 to 300 µg/mL. A mixture of 100 µL of each diluted CSE and 100 µL of the diluted DPPH solution was incubated in the dark at 37 °C for 30 min. After the reaction, absorbance was measured at 517 nm using a microplate spectrophotometer (BioTek Synergy H1) to calculate the remaining DPPH radical levels. For the control group, 100 µL of 100% methanol was used instead of the CSE. The remaining DPPH levels were expressed as percentages (%). AA was used as the positive control.

### 2,2’-azino-bis (3-ethylbenzothiazoline-6-sulfonic acid) diammonium salt (ABTS) assay

ABTS (7.4 mM) and potassium persulfate (2.6 mM) were diluted in distilled water, mixed in 1:1, and reacted in the dark for 16–24 h to produce ABTS^+^. Subsequently, it was diluted with distilled water to give an absorbance of 0.74 ± 0.02 at 734 nm. In a 96-well plate, 100 µL of diluted ABTS^+^ solution and 100 µL of sample were mixed and the absorbance was measured immediately at 734 nm using a microplate spectrometer (Agilent, USA, BioTek Synergy H1). Distilled water was used as control instead of sample and the remaining ABTS was expressed as a percentage (%). AA was used as a positive control.

### UPLC analysis conditions

The extract contents of C, ECG, EGCG, EGC, EC, and CF were determined by comparing UPLC retention times to known standards. Standards were analyzed at 100, 50, 25, 12.5, 6.25, and 3.125 ppm to construct calibration curves for quantitative evaluation. Briefly, 70% ethanol solution of CSE powder was prepared and analyzed using an Agilent 1,290 UPLC system (Agilent, Palo Alto, CA, USA). The chromatographic methods and settings for identifying compounds in CSE are shown in Table 4.

**Table 4:** Separation conditions of UPLC in CSE.

Item	Conditions					
Column	Phenomenex Kinetex C18, 5 μm, 250 × 4.6 mm					
Mobile phase	Time		2 min	40 min	42 min	44 ~ 47 min
	A	0.2% H_3_PO_4_ in water	85%	70%	0%	85%
	B	Acetonitrile	15%	30%	100%	15%
Flow rate	1.0 mL/min					
Column oven temperature.	40 °C					
Detector	UV 240 nm					
Retention time	42 min					

### Molecular docking analysis

Molecular docking analysis was conducted using the UCSF Chimera and AutoDock Vina programs with default settings. The NF-кB protein structure (ID: 4Q3J) was retrieved from the Protein Data Bank and the 3D structures of epigallocatechin compound ID ((CID): 72,277), catechin (CID: 73,160), CF (CID: 2,519), EC (CID: 72,276), EGCG (CID: 65,064), ECG (CID: 107,905), and CPUY192018 (CID: 73,330,369) from PubChem. The results were visualized using Discovery Studio. Binding affinities were determined based on total intermolecular energy and estimated free-energy binding.

### Statistical analysis

GraphPad Prism version 8.0 was used for all statistical analyses (GraphPad Software, SanDiego, CA, USA). One-way factorial analysis of variance was used to analyze the difference. The results are expressed as mean ± SEM or SD. A p-value of < 0.05 was considered statistically significant for all tests.

## Conclusions

We demonstrate that an aqueous extract of *Camellia sinensis* L. (green tea) can suppress the TNF-α/IFN-γ-induced inflammatory response of HaCaT cells, a widely studied in vitro model of AD. CSE exhibited anti-inflammatory properties by reducing the phosphorylation of MAPKs and by limiting the translocation of NF-κB and STAT-1 into the cell nucleus. In addition, we observed that the stimulation of TNF-α and IFN-γ leads to a notable increase in the phosphorylation levels of p38, JNK, and ERK. CSE suppressed the activation of MAPKs, namely p38, JNK, and ERK, as well as the phosphorylation of NF-κB and STAT-1. This finding indicates that CSE possesses anti-inflammatory and antioxidant properties. Therefore, these results suggest that CSE can be used as a potential treatment for inflammatory skin diseases such as AD.

## Electronic supplementary material

Below is the link to the electronic supplementary material.

Supplementary Material 1

## Data Availability

All data generated or analyzed during this study are included in this published article.

## References

[1] Ryu, A. R. & Lee, M. Y. Ameliorative effect of chlorin e6-mediated photodynamic therapy on DNCB-induced atopic dermatitis-like skin lesions in mice. *Mol. Cell. Toxicol.* **15**, 265–270 (2019).

[2] Hadi, H. A. et al. The epidemiology and global burden of atopic dermatitis: a narrative review. *Life (Basel Switzerland)*. **11**10.3390/life11090936 (2021). 10.3390/life11090936 PMC8470589 34575085

[3] Jiang, Y. et al. Cytokinocytes: the diverse contribution of keratinocytes to immune responses in skin. *JCI Insight*. **5**10.1172/jci.insight.142067 (2020). 10.1172/jci.insight.142067 PMC7605526 33055429

[4] Jeong, S. et al. Combined treatment of ginsenoside Rg2 and piceatannol mixture reduces the apoptosis and DNA damage induced by UVB in HaCaT cells. *Mol. Cell. Toxicol.* **19**, 63–70 (2023).

[5] Lee, K. S. et al. The prevention of TNF-alpha/IFN-gamma mixture-induced inflammation in human keratinocyte and atopic dermatitis-like skin lesions in Nc/Nga mice by mineral-balanced deep sea water. *Biomed. Pharmacother.* **97**, 1331–1340. 10.1016/j.biopha.2017.11.056 (2018). 29156522 10.1016/j.biopha.2017.11.056

[6] Medeiros, R., Figueiredo, C. P., Passos, G. F. & Calixto, J. B. Reduced skin inflammatory response in mice lacking inducible nitric oxide synthase. *Biochem. Pharmacol.* **78**, 390–395 (2009). 19409374 10.1016/j.bcp.2009.04.021

[7] Bayazid, A. B. & Jang, Y. A. The role of andrographolide on skin inflammations and modulation of skin barrier functions in human keratinocyte. *Biotechnol. Bioprocess Eng.* **26**, 804–813 (2021).

[8] Cacheiro-Llaguno, C. et al. Regulation of cyclooxygenase-2 expression in human T cells by glucocorticoid receptor-mediated transrepression of nuclear factor of activated T cells. *Int. J. Mol. Sci.* **23**10.3390/ijms232113275 (2022). 10.3390/ijms232113275 PMC9653600 36362060

[9] Bhosale, P. B. et al. Iridin abrogates LPS-induced inflammation in L6 skeletal muscle cells by inhibiting NF-κB and MAPK signaling pathway. *Mol. Cell. Toxicol.* **19**, 483–490 (2023).

[10] Zhang, W. & Liu, H. T. MAPK signal pathways in the regulation of cell proliferation in mammalian cells. *Cell Res.* **12**, 9–18 (2002). 11942415 10.1038/sj.cr.7290105

[11] Lin, Z. M. et al. Topical administration of reversible SAHH inhibitor ameliorates imiquimod-induced psoriasis-like skin lesions in mice via suppression of TNF-α/IFN-γ-induced inflammatory response in keratinocytes and T cell-derived IL-17. *Pharmacol. Res.* **129**, 443–452 (2018). 29155016 10.1016/j.phrs.2017.11.012

[12] Cargnello, M. & Roux, P. P. Activation and function of the MAPKs and their substrates, the MAPK-activated protein kinases. *Microbiol. Mol. Biol. Rev.* **75**, 50–83. 10.1128/MMBR.00031-10 (2011). 21372320 10.1128/MMBR.00031-10 PMC3063353

[13] Yu, Q. et al. Resokaempferol-mediated anti-inflammatory effects on activated macrophages via the inhibition of JAK2/STAT3, NF-κB and JNK/p38 MAPK signaling pathways. *Int. Immunopharmacol.* **38**, 104–114 (2016). 27261558 10.1016/j.intimp.2016.05.010

[14] Kang, Y. M., Kim, H. M., Lee, M. & An, H. J. Oleanolic acid alleviates atopic dermatitis-like responses in vivo and in vitro. *Int. J. Mol. Sci.* **22**10.3390/ijms222112000 (2021). 10.3390/ijms222112000 PMC8584529 34769428

[15] Dias, D. A., Urban, S. & Roessner, U. A historical overview of natural products in drug discovery. *Metabolites* **2**, 303–336. 10.3390/metabo2020303 (2012). 24957513 10.3390/metabo2020303 PMC3901206

[16] Koch, W., Zagorska, J., Marzec, Z. & Kukula-Koch, W. Applications of tea (Camellia sinensis) and its active constituents in cosmetics. *Molecules* **24**10.3390/molecules24234277 (2019). 10.3390/molecules24234277 PMC6930595 31771249

[17] Aboulwafa, M. M. et al. A comprehensive insight on the health benefits and phytoconstituents of Camellia sinensis and recent approaches for its quality control. *Antioxidants* **8**, 455 (2019). 31590466 10.3390/antiox8100455 PMC6826564

[18] Yang, J. H., Hwang, Y. H., Gu, M. J., Cho, W. K. & Ma, J. Y. Ethanol extracts of Sanguisorba officinalis L. suppress TNF-α/IFN-γ-induced pro-inflammatory chemokine production in HaCaT cells. *Phytomedicine* **22**, 1262–1268. 10.1016/j.phymed.2015.09.006 (2015). 26655409 10.1016/j.phymed.2015.09.006

[19] Kang, G. J. et al. Prunus yedoensis inhibits the inflammatory chemokines, MDC and TARC, by regulating the STAT1-signaling pathway in IFN-γ-stimulated HaCaT human keratinocytes. *Biomolecules Ther.* **16**, 394–402 (2008).

[20] Boguniewicz, M. & Leung, D. Y. Atopic dermatitis: a disease of altered skin barrier and immune dysregulation. *Immunol. Rev.* **242**, 233–246. 10.1111/j.1600-065X.2011.01027.x (2011). 21682749 10.1111/j.1600-065X.2011.01027.x PMC3122139

[21] Bieber, T. Atopic dermatitis: an expanding therapeutic pipeline for a complex disease. *Nat. Rev. Drug Discovery*. **21**, 21–40 (2022). 34417579 10.1038/s41573-021-00266-6 PMC8377708

[22] Werfel, T. The role of leukocytes, keratinocytes, and allergen-specific IgE in the development of atopic dermatitis. *J. Invest. Dermatology*. **129**, 1878–1891 (2009). 10.1038/jid.2009.71 19357709

[23] Park, H. J., Jang, Y. J., Yim, J. H., Lee, H. K. & Pyo, S. Ramalin isolated from ramalina terebrata attenuates atopic dermatitis-like skin lesions in balb/c mice and cutaneous immune responses in keratinocytes and mast cells. *Phytother. Res.* **30**, 1978–1987 (2016). 27558640 10.1002/ptr.5703

[24] Sung, Y. Y., Kim, Y. S. & Kim, H. K. Illicium verum extract inhibits TNF-α-and IFN-γ-induced expression of chemokines and cytokines in human keratinocytes. *J. Ethnopharmacol.* **144**, 182–189 (2012). 22974545 10.1016/j.jep.2012.08.049

[25] Yang, J. H. et al. Jageum-Jung improves 2, 4-dinitrochlorobenzene-induced atopic dermatitis-like skin lesions in mice and suppresses pro-inflammatory chemokine production by inhibiting TNF-α/IFN-γ-induced STAT-1 and NFκB signaling in HaCaT cells. *J. Ethnopharmacol.* **221**, 48–55 (2018). 29660465 10.1016/j.jep.2018.04.016

[26] Oh, J. H. et al. Purpurin suppresses atopic dermatitis via TNF-alpha/IFN-gamma-induced inflammation in HaCaT cells. *Int. J. ImmunoPathol Pharmacol.* **36**, 3946320221111135. 10.1177/03946320221111135 (2022). 35794850 10.1177/03946320221111135 PMC9274433

[27] Sharifi-Rad, J. et al. Phenolic compounds as Nrf2 inhibitors: potential applications in cancer therapy. *Cell. Communication Signal.* **21**10.1186/s12964-023-01109-0 (2023). 10.1186/s12964-023-01109-0 PMC10152593 37127651

[28] Waterhouse, A. L. Determination of total phenolics. *Current protocols in food analytical chemistry* 6, I1. 1.1-I1. 1.8 (2002).

[29] Aiyegoro, O. A. & Okoh, A. I. Preliminary phytochemical screening and in vitro antioxidant activities of the aqueous extract of Helichrysum longifolium DC. *BMC Complement. Med. Ther.* **10**10.1186/1472-6882-10-21 (2010). 10.1186/1472-6882-10-21 PMC2877649 20470421

