# Supplementary material for: Anti-inflammatory and antioxidant properties of Camellia sinensis L. extract as a potential therapeutic for atopic dermatitis through NF-κB pathway inhibition
Source: Sci Rep. 2025 Jan 18;15:2371. doi: 10.1038/s41598-025-86678-5 (PMC11742993; doi:10.1038/s41598-025-86678-5)
Supplement: Supplementary file 1 — Supplementary Material 1 [file 41598_2025_86678_MOESM1_ESM.pdf]

Original blots are presented in Supplementary Figure. 3A

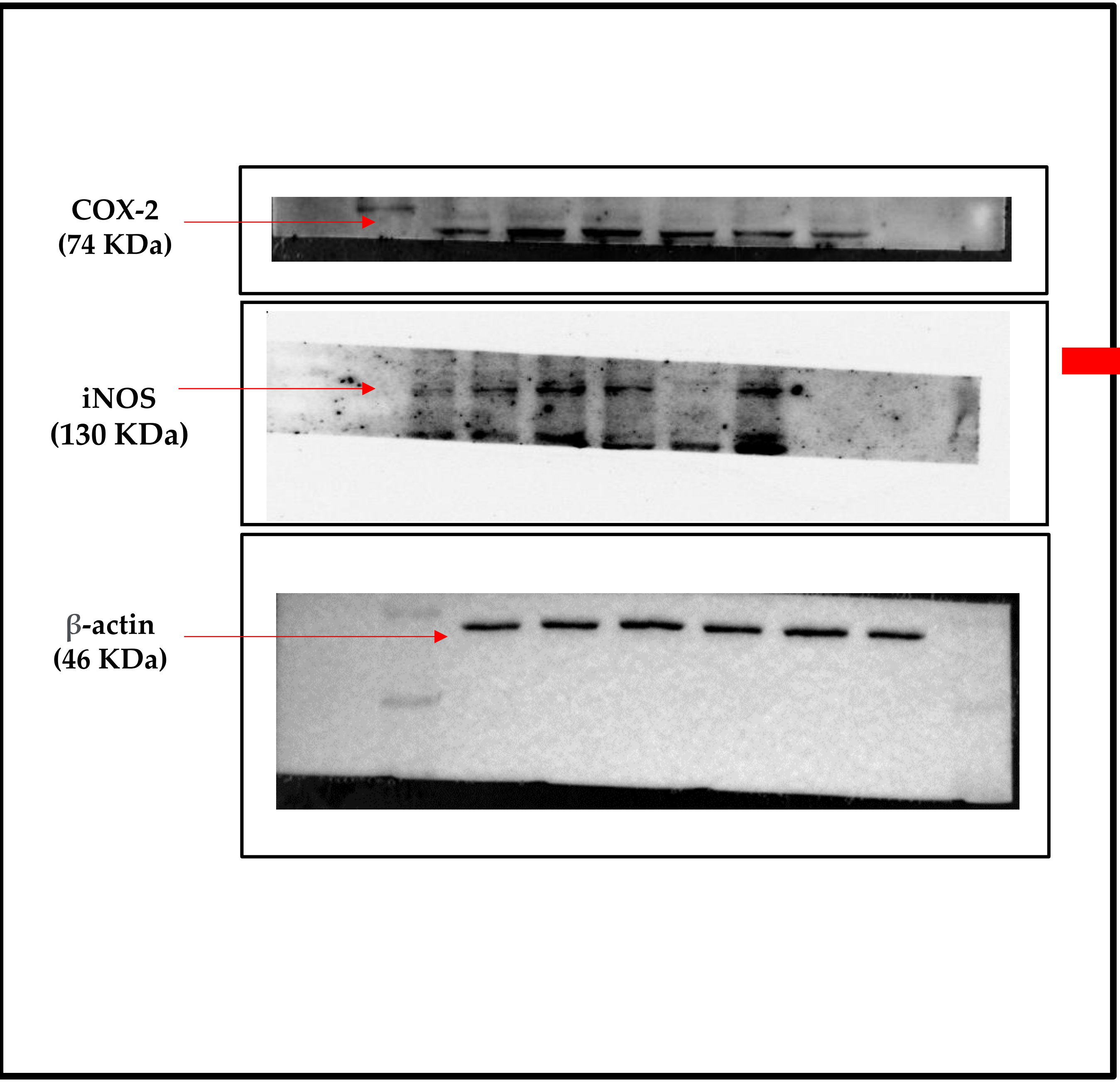

Attached Original Full Blots Images

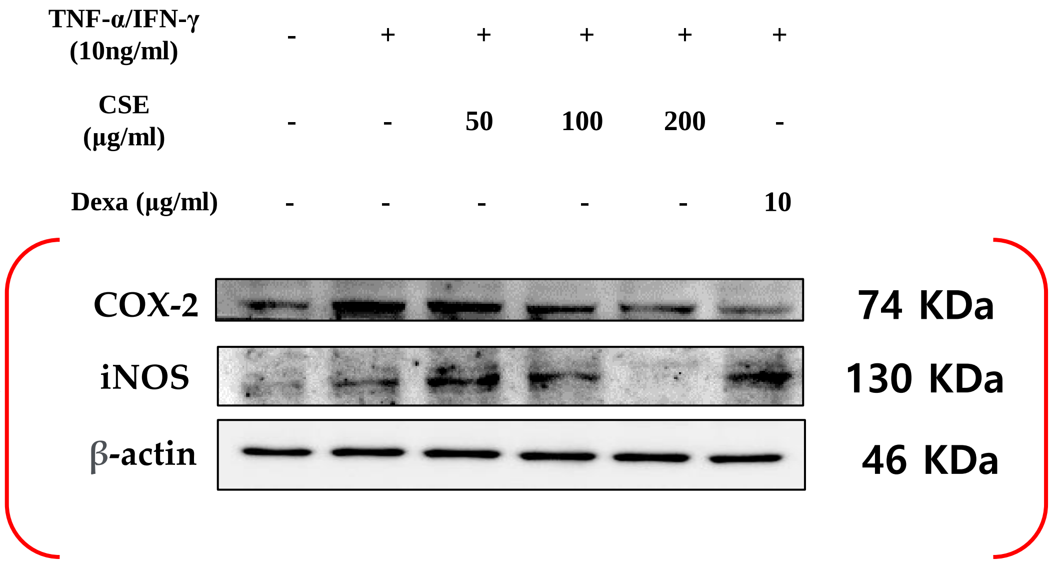

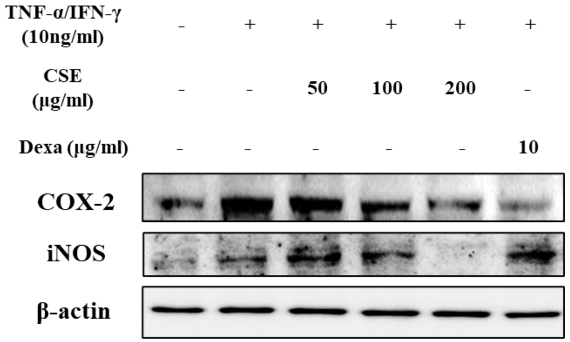

Placed in Figure S3 (A)

Original blots are presented in Supplementary Figure. 4A

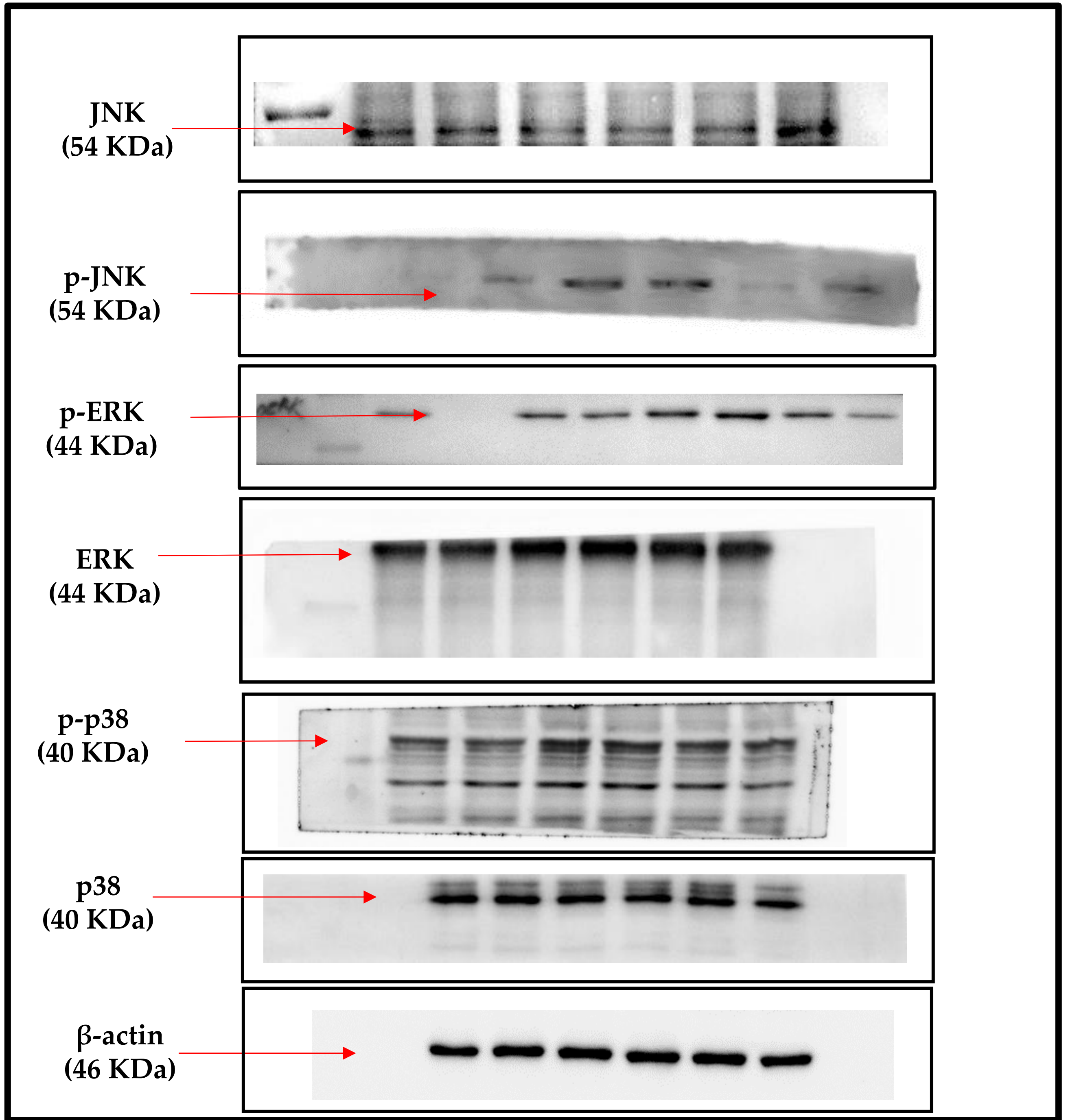

Attached Original Full Blots Images

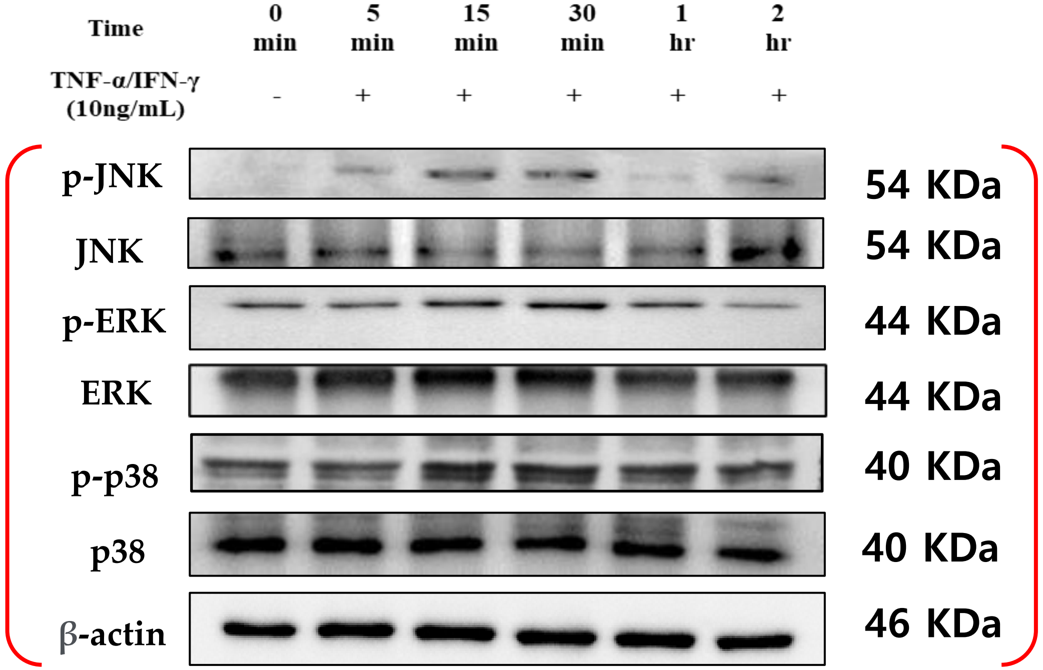

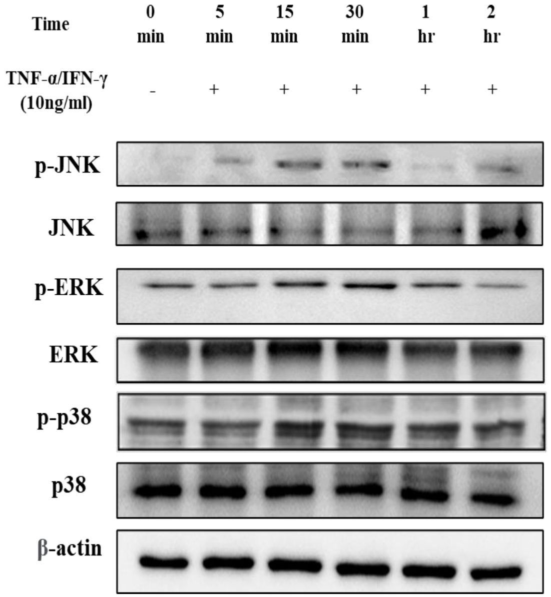

Placed in Figure S4 (A)

Original blots are presented in Supplementary **Figure. 4B**

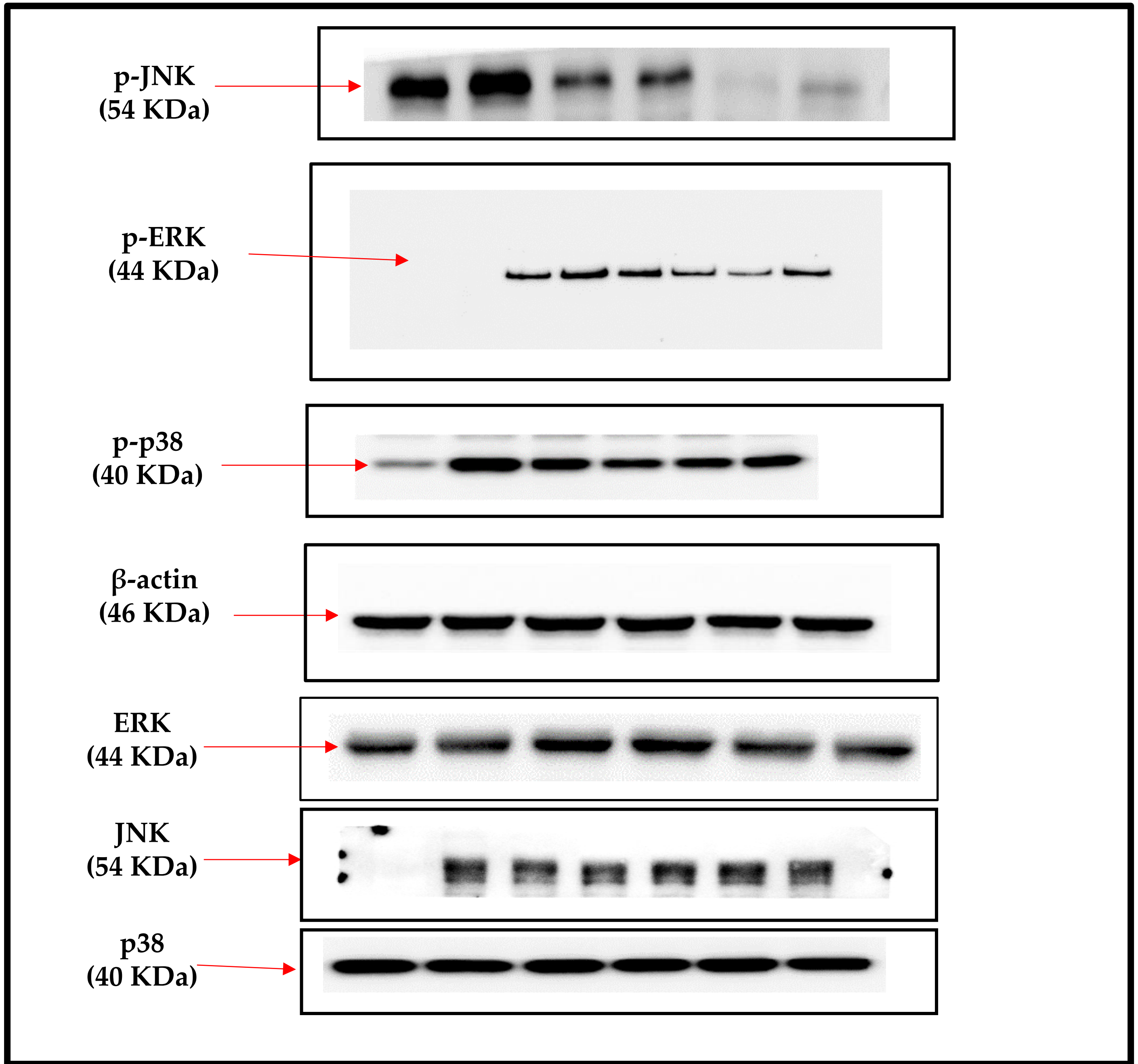

Attached Original Full Blots Images

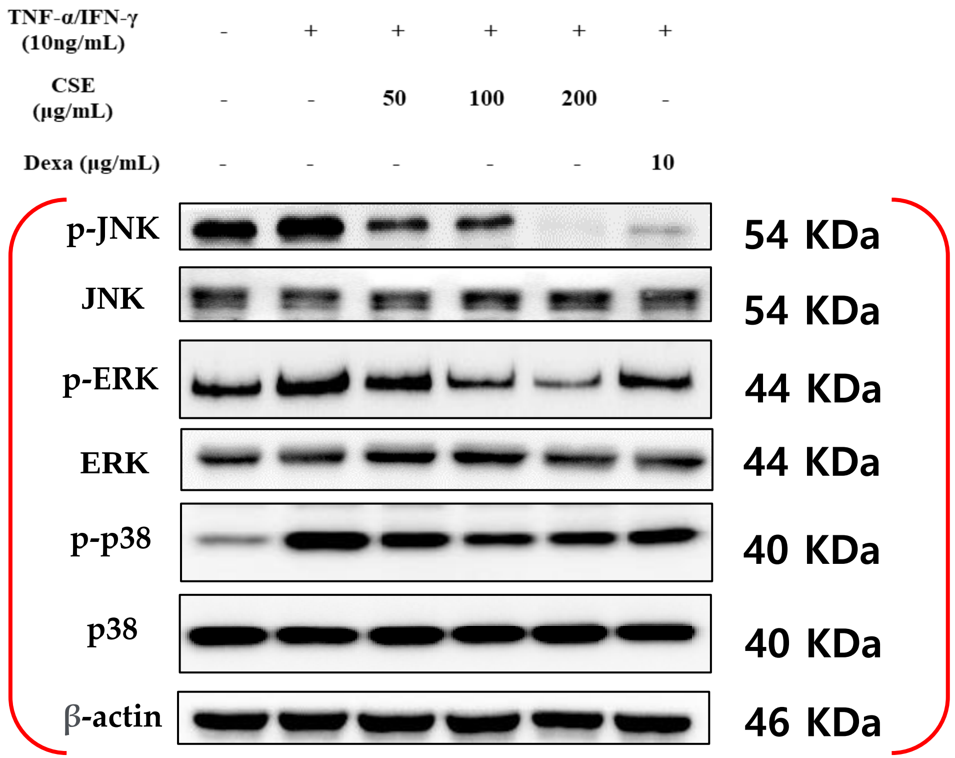

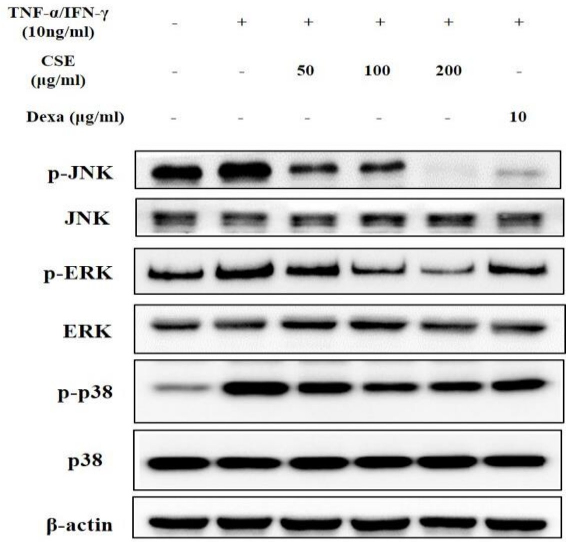

Placed in Figure S4 (B)

Original blots are presented in Supplementary **Figure. 5A**

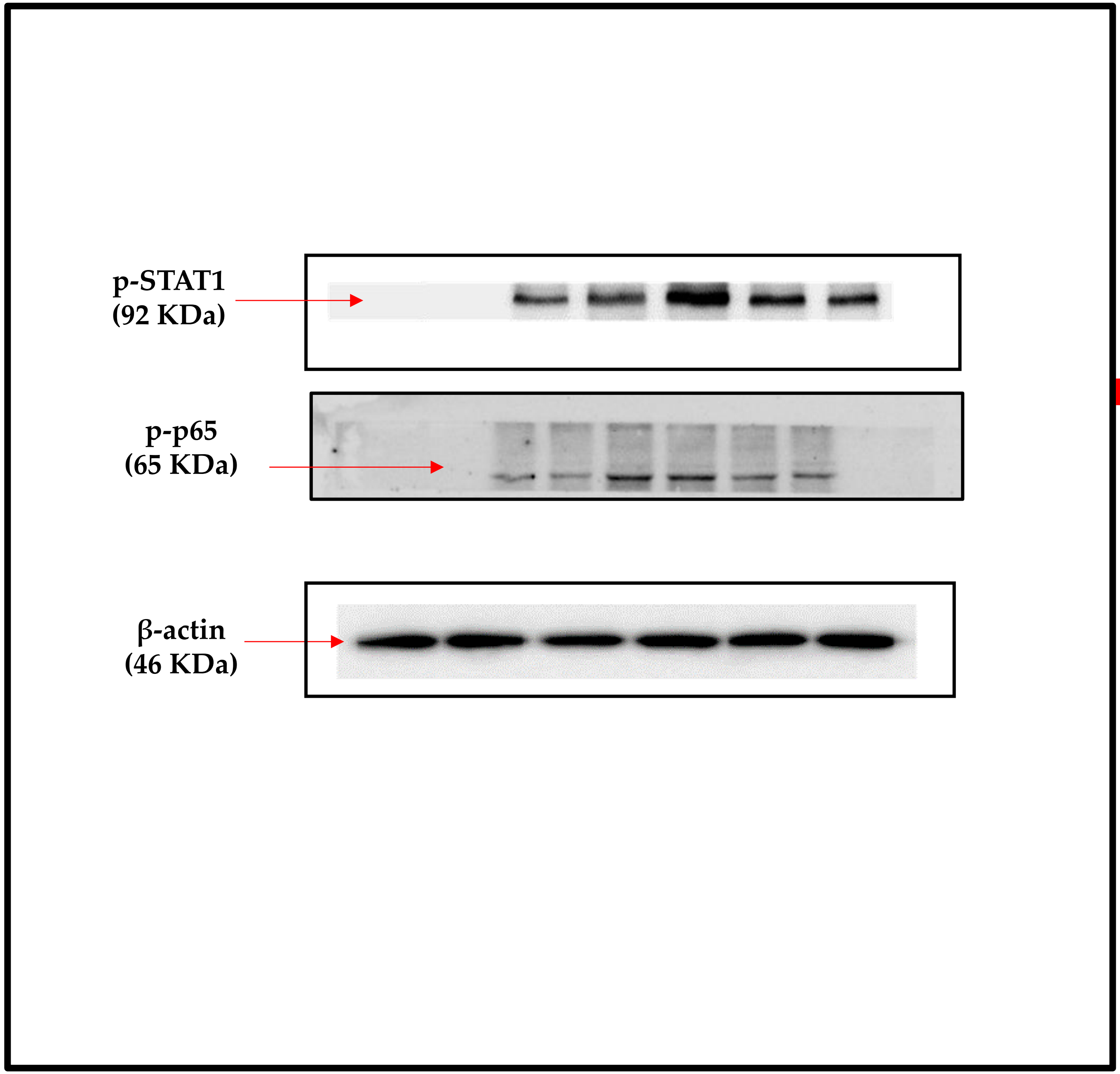

Attached Original Full Blots Images

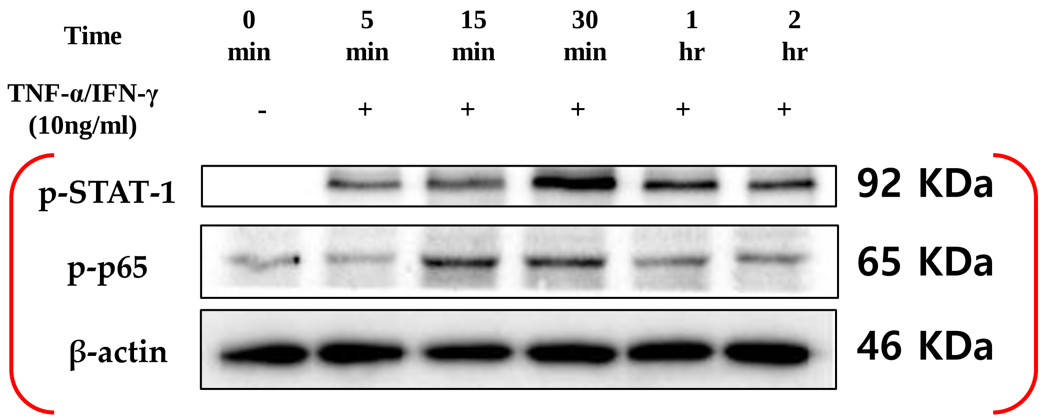

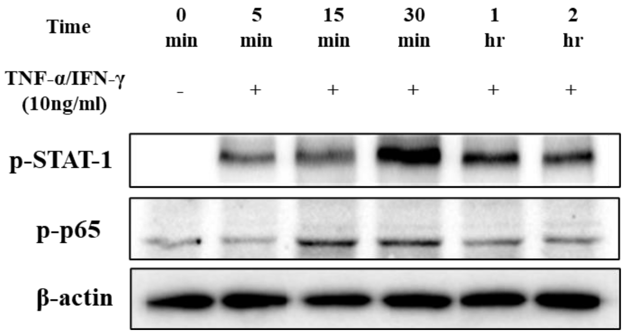

Placed in Figure S5 (A)

Original blots are presented in Supplementary Figure. 5B

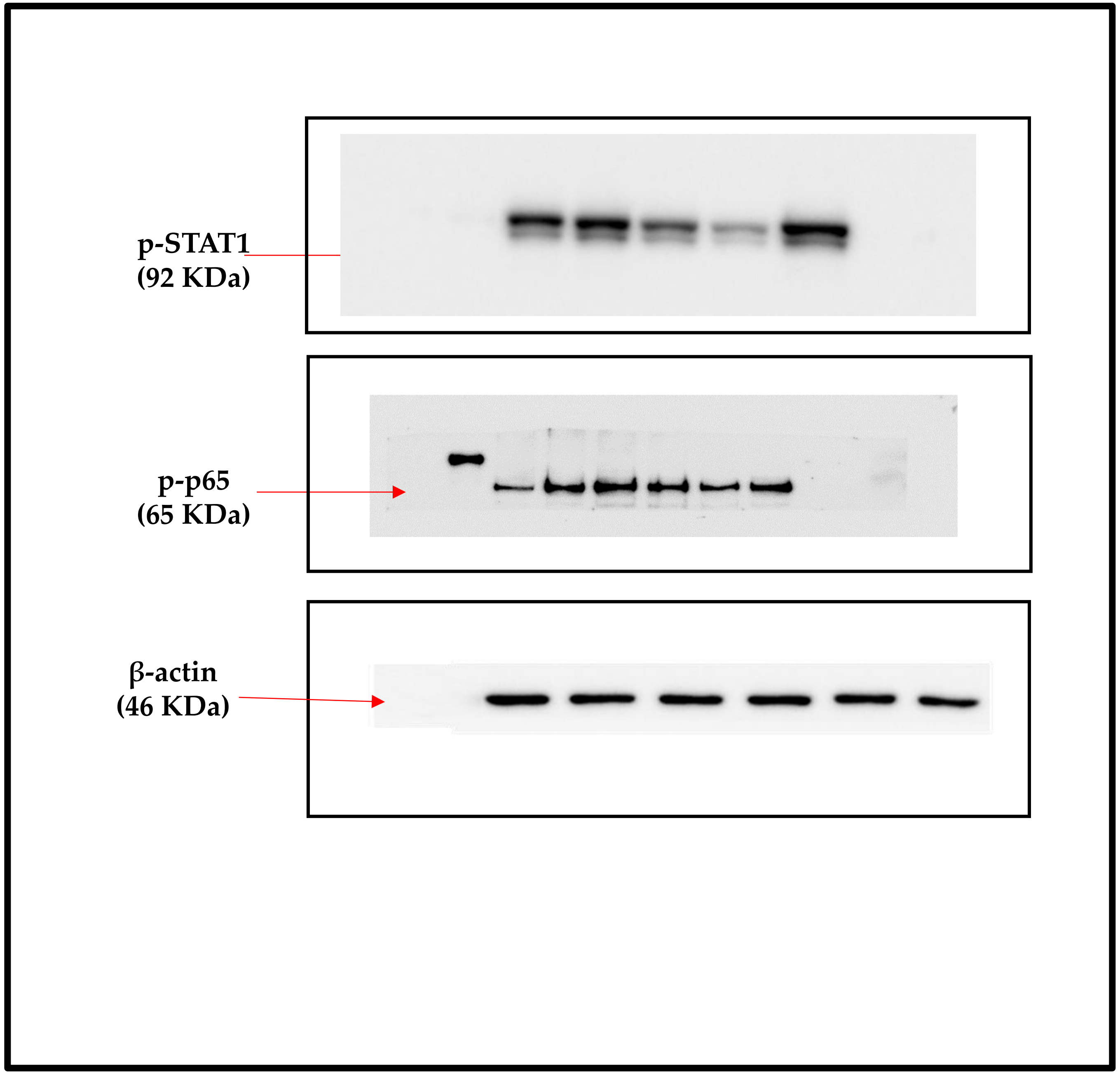

Attached Original Full Blots Images

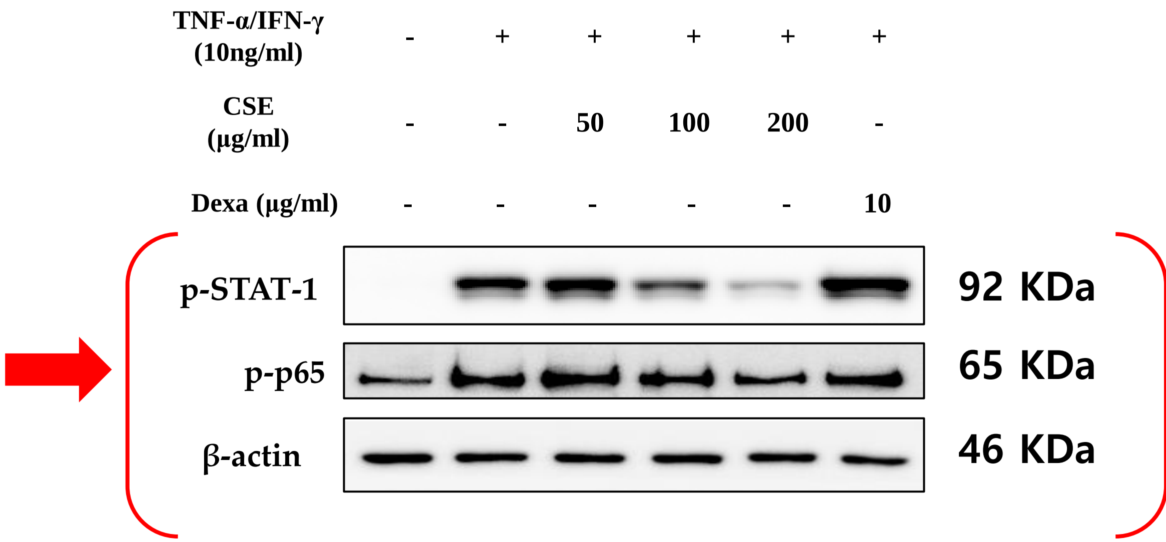

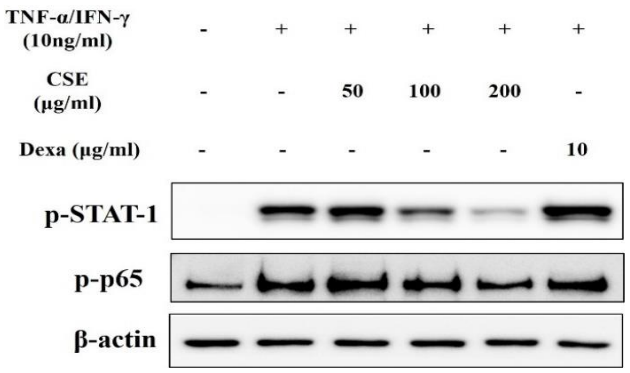

Placed in Figure S5 (B)
